# Therapeutic effects of teriparatide on subchondral bone lesions and pain in mono-iodoacetate-induced osteoarthritis rat model

**DOI:** 10.1016/j.ocarto.2025.100655

**Published:** 2025-07-24

**Authors:** Koji Aso, Natsuki Sugimura, Masashi Izumi, Ikeuchi Masahiko

**Affiliations:** Department of Orthopedic Surgery, Kochi Medical School, Kochi University, 185-1 Oko-cho Kohasu, Nankoku 783-8505, Japan

**Keywords:** Knee osteoarthritis, Pain, Teriparatide, Subchondral bone

## Abstract

**Objectives:**

Knee osteoarthritis (OA) represents a leading cause of chronic pain, with subchondral bone marrow lesions recognized as a critical contributor. Teriparatide (TPTD), a treatment for osteoporosis, promotes subchondral bone remodeling. However, its effects on subchondral bone lesions and associated pain in OA remain unclear. Thus, we aimed to evaluate the therapeutic effects of TPTD in a rat model of monoiodoacetate-induced (MIA)-induced OA.

**Methods:**

Male Sprague-Dawley rats were divided into TPTD ​+ ​MIA, saline ​+ ​MIA, and control groups. OA was induced through intra-articular injection of MIA (1 ​mg). TPTD (30 ​μg/kg) or saline was administered subcutaneously three times per week for 12 weeks. Subchondral bone integrity was assessed by micro-computed tomography imaging. Histological scoring of the cartilage, subchondral bone, and synovium was performed after 12 weeks of treatment. Pain-related behavior was assessed based on hind paw weight distribution and mechanical sensitivity of the hind paw and knee joint.

**Results:**

TPTD preserved subchondral bone integrity, significantly improving bone volume fraction and mineral density. Histological scores for calcified cartilage and subchondral bone damage, and osteoarthritis bone score were reduced; however, no significant differences were observed in cartilage degeneration or synovial inflammation. TPTD administration improved asymmetric weight distribution in advanced OA, although mechanical hyperalgesia in the knee and hind paws remained unchanged. Subchondral bone pathology scores were significantly correlated with asymmetric weight distribution.

**Conclusion:**

TPTD attenuated subchondral bone lesions and improved weight-bearing function in MIA-induced OA, highlighting its therapeutic potential in OA-related pain.

## 1 Introduction

Knee pain is the predominant symptom and the primary driver for seeking medical intervention in patients with knee osteoarthritis (OA). Emerging clinical evidence [[1], [2], [3]] underscores the pivotal role of the subchondral bone in the pathogenesis of OA-related joint pain. Subchondral bone marrow lesions (BMLs), detected via MRI, are significantly more prevalent in individuals with knee OA than in non-arthritis controls [3] and are strongly associated with pain [4]. Furthermore, larger baseline subchondral BMLs have been associated with greater baseline knee pain, whereas an increase in total subchondral BML volume over time has been correlated with worsening knee pain intensity [1]. The presence and size of BMLs have been identified as predictive markers for the future onset and progression of knee pain in OA [5]. Our previous study also found a significant association between the size of subchondral BMLs within the medial femorotibial joint compartment and the severity of weight-bearing pain in patients with varus knee OA. This association was distinct from non-weight-bearing pain and remained significant even after adjusting for other OA-related MRI findings, including effusion synovitis, Hoffa's synovitis, cartilage defects, osteophytes, meniscal extrusions, and anterior cruciate ligament tears [6].

Teriparatide (TPTD, the 1–34 fragment of the human parathyroid hormone) is a key regulator of calcium homeostasis, primarily through its direct action on bone tissue. TPTD has been widely used in the treatment of osteoporosis and bone fractures. It may exert anabolic effects on chondrocytes by enhancing cartilage regeneration [7]. In a mouse model of OA induced by destabilization of the medial meniscus, the progression of OA was effectively prevented by TPTD through the attenuation of cartilage degeneration and the amelioration of abnormal subchondral bone remodeling [8]. Furthermore, TPTD treatment suppresses the onset of back pain and modulates the exacerbation of symptoms [9,10].

Mono-sodium iodoacetate (MIA) injection into the rat knee joint disrupts chondrocyte metabolism, leading to cell death and subsequent loss of articular cartilage accompanied by subchondral bone alterations [11]. The pattern of joint damage observed in the MIA model closely resembles that observed in humans [12,13]. Although BMLs in the subchondral bone of rat OA models are difficult to evaluate due to their small size, subchondral pathologies consistent with those reported in human OA-related BMLs have been identified in the MIA model [14]. This model demonstrates a distinct association between cartilage degeneration and subchondral bone changes, making it a valuable tool for investigating the role of subchondral bone in OA progression [13]. The administration of TPTD three times per week, a regimen used for the treatment of osteoporosis, enhanced the vertebral and peripheral bone density and microarchitecture in ovariectomized rats [15]. However, despite multiple clinical reports highlighting its effects on back pain, the effects of TPTD on pain and subchondral bone lesions in patients with knee OA remain unclear. Therefore, this study aimed to evaluate the effects of weekly TPTD administration on pain and subchondral bone lesions in rats with MIA-induced OA. We hypothesized that TPTD may possess therapeutic potential for alleviating knee OA pain by targeting subchondral bone lesions.

## 2 Methods

### 2.1 Ethics

All experimental procedures were approved by the Animal Care and Use Committee of Kochi University (IRB: H29-088) and conducted in accordance with the Animal Research: Reporting of In Vivo Experiments guidelines.

### 2.2 Animals

A total of 22 male Sprague-Dawley rats (6 weeks old, weight: 250–300 ​g) were used in this study. Prior to the experiments, all animals were acclimatized to the laboratory environment for 1 week. The rats were randomly assigned to one of the three groups (TPTD ​+ ​MIA, saline ​+ ​MIA, and control) using a computer-generated randomization program and mixed within cages. All rats were maintained under standard, environmentally controlled conditions (artificial 12-h light/dark cycle, an ambient temperature of 23 ​± ​1 ​°C, and a relative humidity of 44 ​%–63 ​%). All outcome measurements were carried out by an experimenter blinded to group allocation.

### 2.3 OA induction

OA was induced by intra-articular injection of MIA (1 ​mg). Briefly, after being anesthetized with sodium pentobarbital (30 ​mg/kg, intraperitoneal injection), the TPTD ​+ ​MIA group and saline ​+ ​MIA group (n ​= ​8 knees of 8 rats for each group) were injected with 1 ​mg MIA dissolved in 25 ​μl of saline (Sigma-Aldrich, St. Louis, MO) using a 27-G needle attached to a Hamilton syringe. The injection was administered into the intra-articular space of the left knee through the patellar ligament. In the control group (n ​= ​6 knees of 6 rats), 25 ​μl of saline alone was injected intra-articularly into the left knee.

### 2.4 Drugs

Due to interspecies differences in bone metabolism, the effective dosage regimen of TPTD required for stimulating bone activity varies among species. Previous studies have demonstrated that the subcutaneous administration of TPTD at a dose of 30 ​μg/kg three times per week effectively increases bone mineral density (BMD) in rats [16,17]. Accordingly, in the present study, the TPTD ​+ ​MIA group received subcutaneous injections of TPTD (parathyroid hormone fragment PTH1-34; 30 ​μg/kg diluted in 1 ​ml saline; Asahi Kasei Pharma Corporation, Tokyo, Japan) three times per week for 12 weeks following the MIA injection. Meanwhile, the saline ​+ ​MIA and control groups received subcutaneous injections of 1 ​ml saline at the same frequency and duration. All animals were euthanized by carbon dioxide overdose at 12 weeks after intra-articular injection.

### 2.5 Micro-CT imaging of knee joints

The regions between the distal growth plate of the femur and the proximate growth plate of the tibia were scanned using a micro-CT system (LaTheta LCT-200; Aloka, Osaka, Japan) with a pixel resolution of 24 ​× ​24 ​μm and a slice thickness of 75 ​μm. The system was operated at 50 ​kV and 0.5 ​mA. Threshold values for tissue segmentation were set at 500 ​mg/cm^3^ for differentiating bone from soft tissue and 1200 ​mg/cm^3^ for distinguishing trabecular from cortical bone, as defined by LaTheta software version 3.3. The volume of interest (VOI; mm^3^) was defined as the subchondral region extending from the distal growth plate of the femur to the proximal growth plate of the tibia. This VOI encompassed the subchondral bone of the femoral condyles and tibial plateaus. Cortical bone and joint space were automatically excluded by the system's analysis software, while ossified portions of the menisci were manually excluded. This analysis was performed using axial images acquired from the micro-CT scans (Supplementary File). Quantitative parameters evaluated included bone volume fraction (bone volume/tissue volume (BV/TV)) (%) and bone mineral density (mg/cm^3^).

### 2.6 Histological evaluation of knee joint

Left knee joints were harvested from all animals following all pain-related behavioral assessments at 12 weeks after intra-articular injection of MIA or saline. The knee joints were fixed in 10 ​% neutral-buffered formalin for 3 days, decalcified in 13 ​% formic acid for 12 days, and subsequently embedded in paraffin. To maintain the knee joint in an approximately fully extended position during fixation, each joint was placed individually into a 1.5-cm diameter Spitz-type tube. For histopathological analysis, 5-μm-thick frontal sections were obtained from the middle third of the medial tibiofemoral joint. The sections were stained with hematoxylin and eosin, Safranin O, and fast green. Calcified cartilage and subchondral bone damage, cartilage degeneration, and synovial membrane inflammation were evaluated according to the Osteoarthritis Research Society International grading system criteria [18] (Supplementary File). Briefly, scores for calcified cartilage damage, subchondral bone damage, and synovial membrane inflammation score were calculated using a numerical scale ranging from 0 (no damage) to 5 (severe damage). For each frontal section, the most severely affected lesion on the tibial plateau was recorded. To assess cartilage degeneration, the tibial plateau in each frontal section was divided into three zones of equal width, and each zone was evaluated and scored from 0 (normal) to 5 (severe degeneration). The total cartilage degeneration score was calculated as the sum of the values obtained from the three zones. The maximum possible cartilage degeneration score was 15. Additionally, the rat Osteoarthritis Bone Score [14], a histological scoring system developed to assess pathological features associated with human BMLs, was applied. Seven histological characteristics—cysts, fibrosis, hypervascularity, cartilage islands, trabecular thickening, loss of tidemark integrity, and inflammatory cell infiltration—were evaluated as either present (1) or absent (0), resulting in a total score ranging from 0 to 7 (Supplementary File). Histological BV/TV was also assessed in the femoral condyles and tibial plateaus to evaluate subchondral bone density. BV/TV was calculated as the percentage of bone area relative to the total subchondral tissue area [19].The histological BV/TV was calculated as the percentage of bone area within the total subchondral bone area. The Osteoarthritis Research Society International histological scores, OABS, and histologic BV/TV were subsequently compared among the TPTD ​+ ​MIA, saline ​+ ​MIA, and control groups.

### 2.7 Pain-related behavior tests

Three distinct measures of pain-related behavior were assessed: changes in hind paw weight distribution, mechanical sensitivity of the hind paw, and mechanical sensitivity of the knee joint. Weight distribution was assessed using a hind paw weight-bearing apparatus (Linton Incapacitance Tester; Linton Instrumentation, Norfolk, UK). The apparatus consisted of two force transducers designed to independently measure the weight borne by each hind paw. The rats were placed on the device with their hind paws centered on the transducers. The percentage of weight distributed to the left (ipsilateral) hind paw was calculated using the following formula: % weight distribution of the left hind paw ​= ​left weight/(left weight ​+ ​right weight) ​× ​100 [20].

Mechanical sensitivity was examined using a series of von Frey filaments. The rats were placed inside a Plexiglas cage positioned on an elevated mesh steel platform. Von Frey filaments of varying bending forces (0.4, 0.6, 1, 1.4, 2, 4, 6, 8, 10, 15, and 26 ​g) were applied to the plantar surface of each hind paw in ascending order. Each filament was applied three times for approximately 2–3 ​s or until a withdrawal response was elicited. Upon withdrawal, the paw was retested with filaments in descending order until no response was observed. The sequence was then repeated in ascending order until a withdrawal response recurred. The bending force required to induce a withdrawal response was recorded three times, and the median value was used as the mechanical threshold of the paw [21]. To assess the mechanical sensitivity of the knee joint, the rats were gently restrained in a glove, and a pair of forceps was applied to the left knee joint. The pressure was gradually increased until a withdrawal response was observed. This knee-squeezing procedure was repeated three times during each testing session. The median value of the applied force (g), measured using a computer-based program, was recorded as the mechanical threshold for the knee joint.

### 2.8 Image analysis

All histological scoring and quantification analyses were performed by a single blinded observer (KA) using an all-in-one fluorescence microscope (BZ-X800; KEYENCE, Japan) equipped with the BZ-X800 Analyzer software. The observer was blinded to the group allocation and experimental details of the histological sections.

### 2.9 Statistical analysis

Quantitative data for BV/TV, bone mineral density, and OA histological scores were analyzed using the Kruskal–Wallis tests followed by the Steel-Dwass post hoc test. Pain-related behavioral outcomes were compared using a two-way analysis of variance with Tukey's post hoc test. The associations between calcified cartilage and subchondral bone damage scores, OA bone scores, and weight-bearing asymmetry were evaluated using Spearman's rank correlation coefficient. With regard to sample size estimation, we assumed a two-tailed Mann–Whitney *U* test with an alpha level of 0.05 and a statistical power of 0.80.The expected effect size (Cohen's d) was calculated to be approximately 3.25, based on the difference in calcified cartilage and subchondral bone damage scores between MIA-induced OA and saline-injected control rat knees observed in our previous study [21]. Because multiple groups were compared using the Kruskal–Wallis test followed by the Steel–Dwass post hoc test, the estimated sample size for the primary pairwise comparison was applied to each group. This calculation indicated that six animals per group would be required to achieve sufficient statistical power. Consequently, eight rats were included in each OA group and six rats in the control group. All statistical analyses were performed using JMP version 10 (SAS Institute, Cary, NC, USA) and SPSS version 26.0 (IBM Corp., Armonk, NY, USA). A p-value of <0.05 was considered significant. Sample size estimation was performed using G∗power software (v3.1.9.7, Heinrich-Heine University, Düsseldorf, Germany).

## 3 Results

### 3.1 Micro-CT imaging of knee joints

**Fig. 1:**
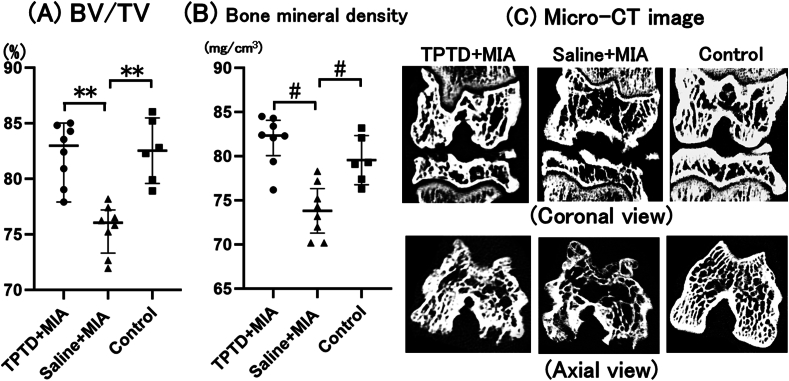
**Micro-CT imaging of the subchondral bone** Each symbol represents an individual sample; bars indicate the median and interquartile range. The BV/TV and BMD were significantly lower in the saline ​+ ​MIA group compared with both the TPTD ​+ ​MIA group (∗∗p ​= ​0.004) and the control group (∗∗p ​= ​0.007). Additionally, the saline ​+ ​MIA group showed lower values compared with the TPTD ​+ ​MIA group (#p ​= ​0.04) and the control group (#p ​= ​0.01). No significant differences were observed between the TPTD ​+ ​MIA and control groups. TPTD: teriparatide, BV/TV: bone volume/tissue volume, MIA: mono-sodium iodoacetate.

Intra-articular injection of MIA into the knee resulted in subchondral bone plate collapse along with irregularities and erosion of the articular bone surfaces (Fig. 1C). Cystic lesions were also observed in the subchondral bone (Fig. 1C). In the saline ​+ ​MIA group, trabecular bone loss was evident, leading to a marked reduction in subchondral bone density. By contrast, TPTD treatment preserved subchondral bone integrity (Fig. 1C). Quantitative analysis demonstrated that both BV/TV and BMD were significantly higher in the TPTD ​+ ​MIA group compared with the saline ​+ ​MIA group (Fig. 1A and B).

### 3.2 Histological analysis of the knee joint

**Fig. 2:**
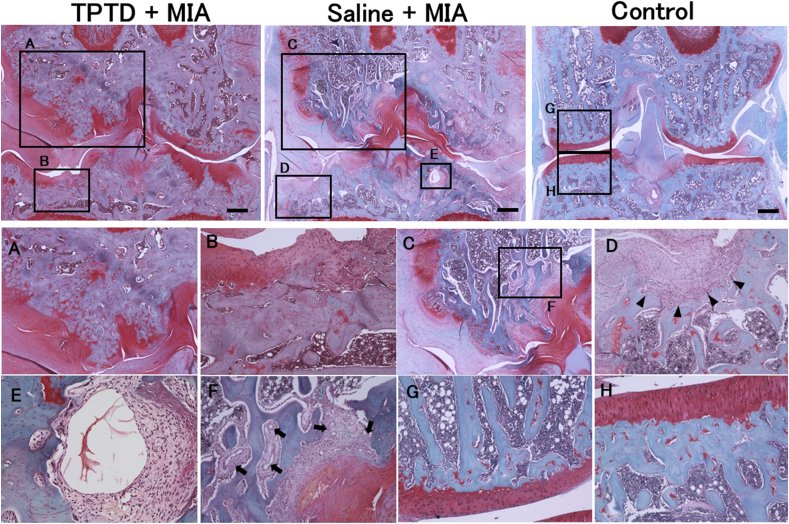
**Histopathologic features of TPTD ​+ ​MIA, saline ​+ ​MIA and control group** Sections were stained with Safranin O and fast green, (Scale bars ​= ​500 ​μm). Severe damage on cartilage surface with loss of proteoglycans was observed in TPTD ​+ ​MIA and saline ​+ ​MIA group. The subchondral bone plate in TPTD ​+ ​MIA group was thicker than saline ​+ ​MIA group (A, B, C and D) and subchondral collapse (arrow heads), cyst and increased vessels (arrows) were observed in saline ​+ ​MIA group (D, E, F). TPTD: teriparatide, MIA: mono-sodium iodoacetate. (For interpretation of the references to colour in this figure legend, the reader is referred to the Web version of this article.)

**Fig. 3:**
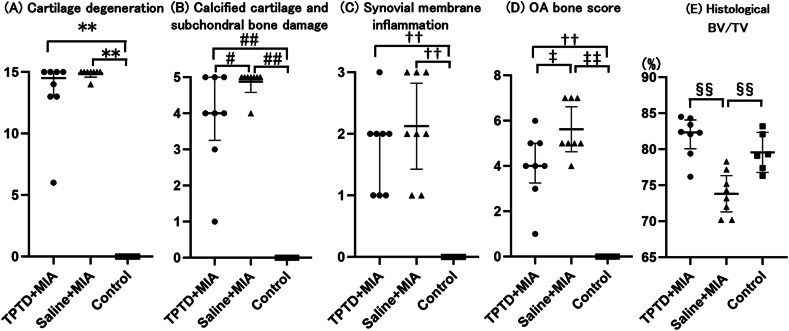
**Cartilage degeneration, calcified cartilage and subchondral bone damage, synovial membrane inflammation score, OABS, and histological BV/TV** Each symbol represents an individual sample; bars indicate the median and interquartile range. Cartilage degeneration, calcified cartilage and subchondral bone damage, synovial membrane inflammation score, and OABS in control group were significantly lower than those in the TPTD ​+ ​MIA and saline ​+ ​MIA group (∗∗p ​= ​0.004 versus TPTD ​+ ​MIA; ∗∗p ​= ​0.002 versus saline ​+ ​MIA group; ##p ​= ​0.001 versus TPTD ​+ ​MIA and saline ​+ ​MIA group; ††p ​= ​0.004 versus TPTD ​+ ​MIA and saline ​+ ​MIA group; ‡‡p ​= ​0.004 versus TPTD ​+ ​MIA and saline ​+ ​MIA group). The calcified cartilage and subchondral bone damage, and OABSs were significantly lower in the TPTD ​+ ​MIA group compared with the saline ​+ ​MIA group (#p ​= ​0.044 versus saline ​+ ​MIA; ‡p ​= ​0.027 versus saline ​+ ​MIA). Histological BV/TV was significantly lower in the saline ​+ ​MIA group compared with the TPTD ​+ ​MIA and control groups (§§p ​= ​0.006 versus the TPTD ​+ ​MIA and §§p ​= ​0.022 control groups). No significant differences were observed between the TPTD ​+ ​MIA and saline ​+ ​MIA groups in cartilage degeneration or synovial membrane inflammation scores. TPTD: teriparatide, MIA: mono-sodium iodoacetate.

Severe damage to the cartilage surface, loss of proteoglycans, and marked subchondral bone damage were induced by intra-articular injection of MIA (Fig. 2A and C). In the saline ​+ ​MIA group, histological changes included subchondral bone fragmentation replaced by fibrous tissue, subchondral collapse, formation of cysts, and increased vascularity within the subchondral bone (Fig. 2C–E). These degenerative changes were markedly attenuated by TPTD treatment, which inhibited the progression of OA-related pathology in the subchondral bone (Fig. 2A and B).The MIA injection significantly increased the scores for cartilage degeneration, calcified cartilage and subchondral bone damage, synovial membrane inflammation, and OA bone (Fig. 3A–D). TPTD treatment significantly improved the scores for calcified cartilage and subchondral bone damage and OA bone, when compared with the saline ​+ ​MIA group (Fig. 3B and D; Table 1; Supplementary Table 1). Histological BV/TV in the TPTD ​+ ​MIA group was significantly higher than that in the saline ​+ ​MIA group (Fig. 3E). However, no significant differences were observed between the TPTD ​+ ​MIA and saline ​+ ​MIA groups in terms of cartilage degeneration and synovial membrane inflammation scores (Fig. 3A and C and Supplementary Table 1).

**Table 1:** Number of rats exhibiting OA bone score features in knee OA models.

	TPTD ​+ ​MIA, n ​= ​8	Saline ​+ ​MIA, n ​= ​8	Control, n ​= ​6
Cysts	1	4	0
Fibrosis	5	7	0
Hypervascularity	4	7	0
Inflammatory cell infiltration	6	8	0
Cartilage islands	4	7	0
Loss of tidemark integrity	3	5	0
Trabecular thickening	8	8	0

Values represent the number of tibial plateaus from each experimental group exhibiting each OA bone score feature. TPTD: teriparatide, MIA: mono-sodium iodoacetate.

### 3.3 Pain-related behavior tests

**Fig. 4:**
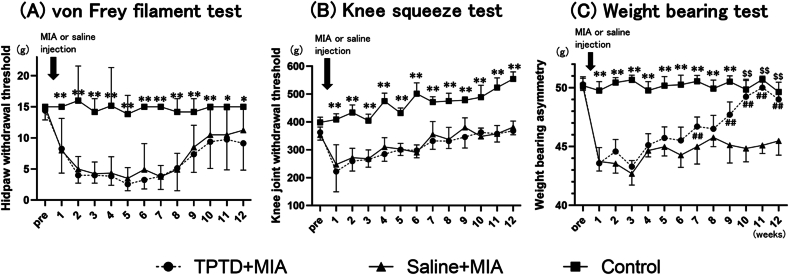
**Effect of teriparatide on mechanical threshold in the knee joint, the hind paw and weight bearing asymmetry of affected hind paw.** Bars show the mean and 95 ​% CI. ∗∗p ​< ​0.001 versus teriparatide ​+ ​MIA and saline ​+ ​MIA group. ##p ​< ​0.001 versus saline ​+ ​MIA group. $$p ​< ​0.001 versus saline ​+ ​MIA group. TPTD: teriparatide, MIA: mono-sodium iodoacetate.

Intra-articular injection of MIA into the knee resulted in mechanical hyperalgesia of the ipsilateral paw and knee joints along with asymmetric weight distribution (Fig. 4A–C).The MIA-induced mechanical hyperalgesia in the paw and knee joint did not occur on the contralateral (right) side. Additionally, the administration of TPTD did not influence pain-related behaviors on the contralateral side (Supplementary Figure). In the saline ​+ ​MIA group, the percentage of weight distribution in the ipsilateral hind paw was reduced from weeks 1–12. By contrast, TPTD treatment significantly inhibited the asymmetric weight distribution induced by MIA during the advanced stages of OA (weeks 7 and 9–12) (Fig. 4C). In both the TPTD ​+ ​MIA and saline ​+ ​MIA groups, progressive mechanical hyperalgesia of the paw and knee was observed from weeks 1–12. However, no significant differences in behavioral test outcomes were detected between the two groups (Fig. 4A and B). With regard to the association between OA histological changes and pain-related behavior, only the OA bone score demonstrated a significant correlation with asymmetric weight distribution (Spearman's r [95 ​% confidence interval] ​= ​−0.63 [−0.889 to −0.129], p ​= ​0.001) (Supplementary Table 2).

## 4 Discussion

This study is the first to demonstrate that three-times-weekly administration of TPTD significantly inhibited subchondral bone lesions and improved asymmetric weight distribution in the hind paw in a rat model of MIA-induced OA. The therapeutic effects of TPTD on weight-bearing asymmetry were associated with a significant reduction in OA bone scores. However, the treatment failed to alleviate hyperalgesia in the knee joint and hind paw.

Subchondral BMLs have previously been reported to be independently associated with weight-bearing pain in patients with knee OA [6]. Therefore, TPTD may alleviate weight-bearing OA pain by suppressing subchondral bone lesions. In the present study, micro-CT imaging demonstrated that TPTD significantly attenuated subchondral bone loss and increased the BV/TV and BMD in the MIA-induced OA model. These findings underscore the potential of TPTD to mitigate pathological alterations in the subchondral bone that are characteristic of OA progression. Subchondral bone lesions are key contributors to OA-related pain, as documented in previous studies [14,22]. Consistent with these findings, the present results suggest that the modulation of subchondral bone remodeling by TPTD may play a pivotal role in reducing weight-bearing pain [23]. Furthermore, as highlighted in a recent systematic review [24], TPTD has demonstrated beneficial effects on subchondral bone microarchitecture and integrity across various animal models of OA. These effects include the prevention of subchondral bone deterioration, enhancement of cartilage metabolism, and suppression of abnormal bone remodeling. Although these preclinical findings are promising, clinical evidence in humans remains limited. Further studies are warranted to determine whether TPTD can effectively target subchondral bone lesions and serve as a disease-modifying treatment for patients with knee OA.

Pain is one of the most debilitating symptoms of knee OA and is a primary reason drives patients seek medical intervention. In the present study, TPTD treatment significantly improved asymmetric weight distribution—a commonly used surrogate indicator of pain—during the advanced stages of OA (9–12 weeks post-induction). This finding is consistent with the report of previous studies indicating that subchondral bone pathology is closely associated with mechanical pain in OA [6,14,25]. TPTD effectively improved the structure of subchondral bone and calcified cartilage; however, it did not exert significant effects on cartilage degeneration or synovial inflammation [22,23]. These findings are consistent with the hypothesis that the therapeutic effects of TPTD in OA are primarily mediated through its action on the subchondral bone. However, the lack of TPTD improvement in mechanical hyperalgesia of the hind paw and knee joint suggests that other pain mechanisms—such as synovial inflammation or central sensitization—may remain unaddressed [23,25]. Both clinical and preclinical studies have indicated that synovitis, rather than subchondral lesions, may play a more central role in driving widespread and sensitized pain in OA [26]. In MIA-induced rat OA models, histological synovitis correlated more strongly with reduced hind paw withdrawal thresholds than with weight-bearing asymmetry [26]. Similarly, in human OA, synovitis has been associated with lower pressure pain thresholds at both local and distant sites, reflecting the contribution of central sensitization [27]. These findings suggest that combination therapies targeting both structural changes and inflammatory processes may be necessary to achieve comprehensive pain control in OA.

The observed effects of TPTD on subchondral bone lesions and weight-bearing OA pain suggest that other osteoporosis treatments, such as bisphosphonates and denosumab, may also offer therapeutic benefits in patients with OA. In a medial meniscal tear model, the early administration of zoledronic acid reduced subchondral bone changes, cartilage degeneration, and pain-related behavior, whereas delayed treatment yielded minimal benefit [28]. Similarly, clodronate administration demonstrated protective effects against cartilage degradation and subchondral bone changes in advanced MIA-induced OA [29]. In a mouse model of OA induced by destabilization of the medial meniscus, denosumab treatment significantly improved subchondral bone structure and suppressed OA progression [30]. In clinical studies involving humans, zoledronic acid reduced the subchondral BML size and alleviated knee pain in individuals with OA [31]. However, a more recent meta-analysis [32] and a randomized controlled trial [33] did not support the consistent analgesic effect of bisphosphonates in the management of knee OA. These conflicting findings highlight the need for further research to explore the effectiveness of osteoporosis medications in the treatment of OA and broaden the scope of innovative treatment options.

This study has some limitations. First, a rat model of OA induced by MIA injection was employed, which may not fully replicate the complexity and gradual progression characteristic of human OA. Although surgically induced models—such as medial meniscal tear, anterior cruciate ligament transection, and destabilization of the medial meniscus—more closely mimic the natural course of human OA, they require longer experimental durations to detect significant structural and pain-related changes. In the present study, the MIA-induced model was selected to allow the evaluation of early therapeutic effects within a shorter timeframe. However, previous studies using surgical models have demonstrated beneficial effects of TPTD. For example, in a DMM mouse model, TPTD inhibited TNF-α expression, suppressed MMP-13 production [34], and attenuated both subchondral bone and cartilage degeneration [8,34]. Similarly, in a guinea pig meniscectomy model, TPTD demonstrated superior efficacy compared with celecoxib in preserving cartilage metabolism and subchondral bone microarchitecture [35]. These findings suggest the importance of future studies employing surgical OA models to validate and extend upon our results in conditions that more closely mimic human OA progression. Second, this study focused primarily on the effects of TPTD on specific histological features of OA—such as subchondral bone lesions and pain-related behaviors. However, potential effects on the central nervous system, including mechanisms of central sensitization, were not assessed. Moreover, the study duration was limited to 12 weeks, and the long-term efficacy and safety of TPTD in OA remain unknown. Long-term follow-up studies are warranted to determine the durability of the therapeutic effects observed.

In conclusion, TPTD demonstrates significant potential as a therapeutic agent for OA, particularly in mitigating subchondral bone lesions and improving weight-bearing pain. These findings provide a strong rationale for further investigation of TPTD in clinical settings and suggest the potential for repurposing other medications for OA treatment. Although additional studies are required to validate these results and address the limitations of the current research, TPTD represents a promising step toward the development of innovative and multifaceted treatment strategies for OA.

## Author contributions

All authors approved the final version of the manuscript for publication. K.A. designed the experiments, analyzed and interpreted the results, and drafted the manuscript. K.A. performed the histological analysis and pain-related behavior tests. N.S. and M.I. contributed to the interpretation of the results. M.I. supervised this study.

## Ethics approval and consent to participate

Thid study was approved by the Kochi University Research Ethics Committee (IRB: H29-088).

## Consent for publication

All authors agreed to the publication of this manuscript.

## Declaration of competing interest

Teriparatide was provided by Asahi Kasei Pharma Corporation.

## References

[1] Driban J.B., Price L., Lo G.H., Pang J., Hunter D.J., Miller E. (2013). Evaluation of bone marrow lesion volume as a knee osteoarthritis biomarker--longitudinal relationships with pain and structural changes: data from the Osteoarthritis Initiative. Arthritis Res. Ther..

[2] Lo G.H., McAlindon T.E., Niu J., Zhang Y., Beals C., Dabrowski C. (2009). Bone marrow lesions and joint effusion are strongly and independently associated with weight-bearing pain in knee osteoarthritis: data from the Osteoarthritis Initiative. Osteoarthr. Cartil..

[3] Stahl R., Jain S.K., Lutz J., Wyman B.T., Le Graverand-Gastineau M.P., Vignon E. (2011). Osteoarthritis of the knee at 3.0 T: comparison of a quantitative and a semi-quantitative score for the assessment of the extent of cartilage lesion and bone marrow edema pattern in a 24-month longitudinal study. Skelet. Radiol..

[4] Walsh D.A., Sofat N., Guermazi A., Hunter D.J. (2023). Osteoarthritis bone marrow lesions. Osteoarthr. Cartil..

[5] Roemer F.W., Collins J.E., Neogi T., Crema M.D., Guermazi A. (2020). Association of knee OA structural phenotypes to risk for progression: a secondary analysis from the Foundation for National Institutes of Health Osteoarthritis Biomarkers study (FNIH). Osteoarthr. Cartil..

[6] Aso K., Shahtaheri S.M., McWilliams D.F., Walsh D.A. (2021). Association of subchondral bone marrow lesion localization with weight-bearing pain in people with knee osteoarthritis: data from the Osteoarthritis Initiative. Arthritis Res. Ther..

[7] Rajagopal K., Ramesh S., Madhuri V. (2021). Early addition of parathyroid hormone-related peptide regulates the hypertrophic differentiation of mesenchymal stem cells. Cartilage.

[8] Li G., Liu S., Chen Y., Xu H., Qi T., Xiong A. (2023). Teriparatide ameliorates articular cartilage degradation and aberrant subchondral bone remodeling in DMM mice. J. Orthop. Translat..

[9] Genant H.K., Halse J., Briney W.G., Xie L., Glass E.V., Krege J.H. (2005). The effects of teriparatide on the incidence of back pain in postmenopausal women with osteoporosis. Curr. Med. Res. Opin..

[10] Nevitt M.C., Chen P., Dore R.K., Reginster J.Y., Kiel D.P., Zanchetta J.R. (2006). Reduced risk of back pain following teriparatide treatment: a meta-analysis. Osteoporos. Int..

[11] van der Kraan P.M., Vitters E.L., van de Putte L.B., van den Berg W.B. (1989). Development of osteoarthritic lesions in mice by "metabolic" and "mechanical" alterations in the knee joints. Am. J. Pathol..

[12] Guingamp C., Gegout-Pottie P., Philippe L., Terlain B., Netter P., Gillet P. (1997). Mono-iodoacetate-induced experimental osteoarthritis: a dose-response study of loss of mobility, morphology, and biochemistry. Arthritis Rheum..

[13] Guzman R.E., Evans M.G., Bove S., Morenko B., Kilgore K. (2003). Mono-iodoacetate-induced histologic changes in subchondral bone and articular cartilage of rat femorotibial joints: an animal model of osteoarthritis. Toxicol. Pathol..

[14] McWilliams D.F., Shahtaheri M., Koushesh S., Joseph C., Gowler P.R., Xu L. (2025). The rat osteoarthritis bone score for histological pathology relevant to human bone marrow lesions and pain. Osteoarthr. Cartil. Open.

[15] Takao-Kawabata R., Isogai Y., Takakura A., Shimazu Y., Sugimoto E., Nakazono O. (2015). Three-times-weekly administration of teriparatide improves vertebral and peripheral bone density, microarchitecture, and mechanical properties without accelerating bone resorption in ovariectomized rats. Calcif. Tissue Int..

[16] Iwamoto J., Seki A. (2015). Effect of combined teriparatide and monthly risedronate therapy on cancellous bone mass in orchidectomized rats: a bone histomorphometry study. Calcif. Tissue Int..

[17] Takakura A., Lee J.W., Hirano K., Isogai Y., Ishizuya T., Takao-Kawabata R. (2017). Administration frequency as well as dosage of PTH are associated with development of cortical porosity in ovariectomized rats. Bone Res..

[18] Gerwin N., Bendele A.M., Glasson S., Carlson C.S. (2010). The OARSI histopathology initiative - recommendations for histological assessments of osteoarthritis in the rat. Osteoarthr. Cartil..

[19] Aso K., Shahtaheri S.M., Hill R., Wilson D., McWilliams D.F., Walsh D.A. (2019). Associations of symptomatic knee osteoarthritis with histopathologic features in subchondral bone. Arthritis Rheumatol..

[20] Aso K., Walsh D.A., Wada H., Izumi M., Tomitori H., Fujii K. (2022). Time course and localization of nerve growth factor expression and sensory nerve growth during progression of knee osteoarthritis in rats. Osteoarthr. Cartil..

[21] Aso K., Izumi M., Sugimura N., Okanoue Y., Ushida T., Ikeuchi M. (2016). Nociceptive phenotype alterations of dorsal root ganglia neurons innervating the subchondral bone in osteoarthritic rat knee joints. Osteoarthr. Cartil..

[22] Orth P., Cucchiarini M., Zurakowski D., Menger M.D., Kohn D.M., Madry H. (2013). Parathyroid hormone [1-34] improves articular cartilage surface architecture and integration and subchondral bone reconstitution in osteochondral defects in vivo. Osteoarthr. Cartil..

[23] Bagi C.M., Berryman E., Zakur D.E., Wilkie D., Andresen C.J. (2015). Effect of antiresorptive and anabolic bone therapy on development of osteoarthritis in a posttraumatic rat model of OA. Arthritis Res. Ther..

[24] Li G., Liu S., Xu H., Chen Y., Deng J., Xiong A. (2023). Potential effects of teriparatide (PTH (1-34)) on osteoarthritis: a systematic review. Arthritis Res. Ther..

[25] Satake Y., Izumi M., Aso K., Igarashi Y., Sasaki N., Ikeuchi M. (2021). Comparison of predisposing factors between pain on walking and pain at rest in patients with knee osteoarthritis. J. Pain Res..

[26] Nwosu L.N., Mapp P.I., Chapman V., Walsh D.A. (2016). Relationship between structural pathology and pain behaviour in a model of osteoarthritis (OA). Osteoarthr. Cartil..

[27] Neogi T., Guermazi A., Roemer F., Nevitt M.C., Scholz J., Arendt-Nielsen L. (2016). Association of joint inflammation with pain sensitization in knee osteoarthritis: the multicenter osteoarthritis study. Arthritis Rheumatol..

[28] Yu D.G., Yu B., Mao Y.Q., Zhao X., Wang X.Q., Ding H.F. (2012). Efficacy of zoledronic acid in treatment of teoarthritis is dependent on the disease progression stage in rat medial meniscal tear model. Acta Pharmacol. Sin..

[29] Sasaki Y., Kijima K., Yoshioka K. (2024). Validity evaluation of a rat model of monoiodoacetate-induced osteoarthritis with clinically effective drugs. BMC Muscoskelet. Disord..

[30] Shangguan L., Ding M., Wang Y., Xu H., Liao B. (2024). Denosumab ameliorates osteoarthritis by protecting cartilage against degradation and modulating subchondral bone remodeling. Regen. Ther..

[31] Laslett L.L., Doré D.A., Quinn S.J., Boon P., Ryan E., Winzenberg T.M. (2012). Zoledronic acid reduces knee pain and bone marrow lesions over 1 year: a randomised controlled trial. Ann. Rheum. Dis..

[32] Vaysbrot E.E., Osani M.C., Musetti M.C., McAlindon T.E., Bannuru R.R. (2018). Are bisphosphonates efficacious in knee osteoarthritis? A meta-analysis of randomized controlled trials. Osteoarthr. Cartil..

[33] Cai G., Laslett L.L., Aitken D., Cicuttini F., March L., Hill C. (2019). Zoledronic acid plus methylprednisolone versus zoledronic acid or placebo in symptomatic knee osteoarthritis: a randomized controlled trial. Ther. Adv. Musculoskelet. Dis..

[34] He Y.J., Liang X., Zhang X.X., Li S.S., Sun Y., Li T.F. (2021). PTH1-34 inhibited TNF-α expression and antagonized TNF-α-induced MMP13 expression in MIO mice. Int. Immunopharmacol..

[35] Dai M.W., Chu J.G., Tian F.M., Song H.P., Wang Y., Zhang Y.Z. (2016). Parathyroid hormone(1-34) exhibits more comprehensive effects than celecoxib in cartilage metabolism and maintaining subchondral bone micro-architecture in meniscectomized Guinea pigs. Osteoarthr. Cartil..

