# Supplementary material for: Therapeutic effects of teriparatide on subchondral bone lesions and pain in mono-iodoacetate-induced osteoarthritis rat model
Source: Osteoarthr Cartil Open. 2025 Jul 24;7(3):100655. doi: 10.1016/j.ocarto.2025.100655 (PMC12344250; doi:10.1016/j.ocarto.2025.100655)
Supplement: Multimedia component 1 [file mmc1.docx]

**SUPPLEMENTARY FILE**

**Supplementary methods**

**Micro-CT analysis of knee joints**

The volume of interest (VOI; mm3) was set as the subchondral bone between the distal growth plate of the femur and the proximate growth plate of the tibia. The VOI included subchondral bone (yellow area) of the femoral condyles and tibial plateaus. Cortical bone (blue area) and joint space were automatically excluded using the analysis software provided with the micro-CT system, while ossified portions of the menisci were manually excluded. This analysis was performed using axial images obtained from the micro-CT scans

**
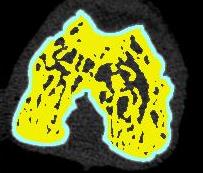
 A representative image of the micro-CT analysis**

**A. Osteoarthritis (OA) Research Society International recommendations for histological assessments of OA in rats**

**#1** **Cartilage degeneration score**- for obtaining the cartilage degeneration score, the medial tibial plateau was divided into 3 zones. Cartilage degeneration in each zone was scored from ‘none’ to ‘severe’ (numerical values 0–5): 0 [No degeneration], 1 [Minimal degeneration; 5%–10% of the total projected cartilage area affected by matrix or chondrocyte loss], 2 [Mild degeneration; 11%–25% affected], 3 [Moderate degeneration; 26%–50% affected], 4 [Marked degeneration; 51%–75% affected], 5 [Severe degeneration; greater than 75% affected]. The sum of cartilage degeneration scores in 3 zones was calculated. The maximum score of cartilage degeneration was 15.

**#2 Calcified cartilage and subchondral bone damage score**- Grade 0 (normal calcified cartilage and subchondral bone; however, a slight increase in basophilia of the calcified cartilage in the central load-bearing area of the joint was seen), Grade 1 (increased basophilia at the tidemark and minimal focal marrow changes. Increased thickening of subchondral bone subjacent to the area of greatest cartilage lesion severity is observed in Grade 1 and all higher grades), Grade 2 (increased basophilia at the tidemark, minimal to mild focal fragmentation of calcified cartilage of the tidemark, and mesenchymal change in marrow involving one-quarter of the subchondral region under lesion), and Grade 3 (increased basophilia at the tidemark, mild to marked multifocal fragmentation of calcified cartilage, and mesenchymal change in the marrow of up to three-quarters of the total area and areas of marrow chondrogenesis are evident), Grade 4 (increased basophilia at the tidemark, marked to severe fragmentation of calcified cartilage, and marrow mesenchymal changes involving up to three-quarters of the area). Articular cartilage collapsed into the epiphysis: see definite depression in surface cartilage. Basophilic areas under the collapsed area result from chondrogenesis in the bone marrow), Grade 5 (marked to severe fragmentation of calcified cartilage and subchondral bone with collapse of cartilage and some chondrogenesis in the marrow. Marrow mesenchymal changes involve up to three-quarters of the area and a large bone cyst is present. Articular cartilage has collapsed into the epiphysis to a depth >250 μm from the tidemark with associated bone resorption). The most severe lesion is scored in each section.

**#3 Synovial membrane inflammation score**- 0 (No changes [1–2 layers of synovial lining cells]), 1 (Increased number of lining cell layers [≥3–4 layers] or slight proliferation of subsynovial tissue), 2 (Increased number of lining cell layers [≥3–4 layers] and/or proliferation of subsynovial tissue), 3 (Increased number of lining cell layers [>4 layers] and/or proliferation of subsynovial tissue and infiltration of few inflammatory cells), 4 (Increased number of lining cell layers [>4 layers] and/or proliferation of subsynovial tissue and infiltration of a large number of inflammatory cells).

**Osteoarthritis Bone Score (OABS) in rat knee subchondral region:- scoring of Present/Absent**

**1. Cysts**

Rounded structures with an outer wall displaying eosinophilic parallel fibres consistent with collagen fibres. Ensure that vessels, adipocytes and sinusoids are not classified as cysts. Scored as present if at least 1 cyst.

**2. Fibrosis**

Eosinophilic parallel fibres consistent with collagen, with or without fusiform mononucleated cells consistent with being fibroblasts. Scored as Present if any bone marrow space displays fibrosis.

**3. Blood vessels**

Annular or tubular structures with cellular (endothelial cell) walls, often have erythrocytes within the lumen and often have smooth muscle around them. Sinusoids are not counted as vessels. Scored as Present if >15 blood vessels within the subchondral region of interest.

**4. Cartilage islands.**

New cartilage (eosinophilic or safranin-O stained matrix containing chondrocytes) that is discontinuous with articular cartilage. Acellular regions of proteoglycan within bone are not counted as cartilage islands. Scored as Present if at least 1 cartilage island with chondrocytes is within the subchondral region of interest.

**5. Trabeculae thickened.**

Scored as present if at least 3 trabeculae show increased thickness (>50 µm at the widest point between junctions with other trabeculae) between the subchondral bone plate and growth plate.

**6. Vessels entering the cartilage**

Scored as Present if at least 1 channel (cellular tissue forming a tubular structure that may contain a blood vessel) enters the articular cartilage. Score as 0 if there is no articular cartilage.

**7. Inflammation**

Non-fusiform mononuclear cells embeded within eosinophilic tissue. Multinucleated cells, consistent with being osteoclasts, and fibroblast-like cells may be present, but should not be the only cell type. Scored as Present if at least 1 area of inflammation.
