# Supplementary material for: Therapeutic effects of teriparatide on subchondral bone lesions and pain in mono-iodoacetate-induced osteoarthritis rat model
Source: Osteoarthr Cartil Open. 2025 Jul 24;7(3):100655. doi: 10.1016/j.ocarto.2025.100655 (PMC12344250; doi:10.1016/j.ocarto.2025.100655)
Supplement: Multimedia component 2 [file mmc2.docx]

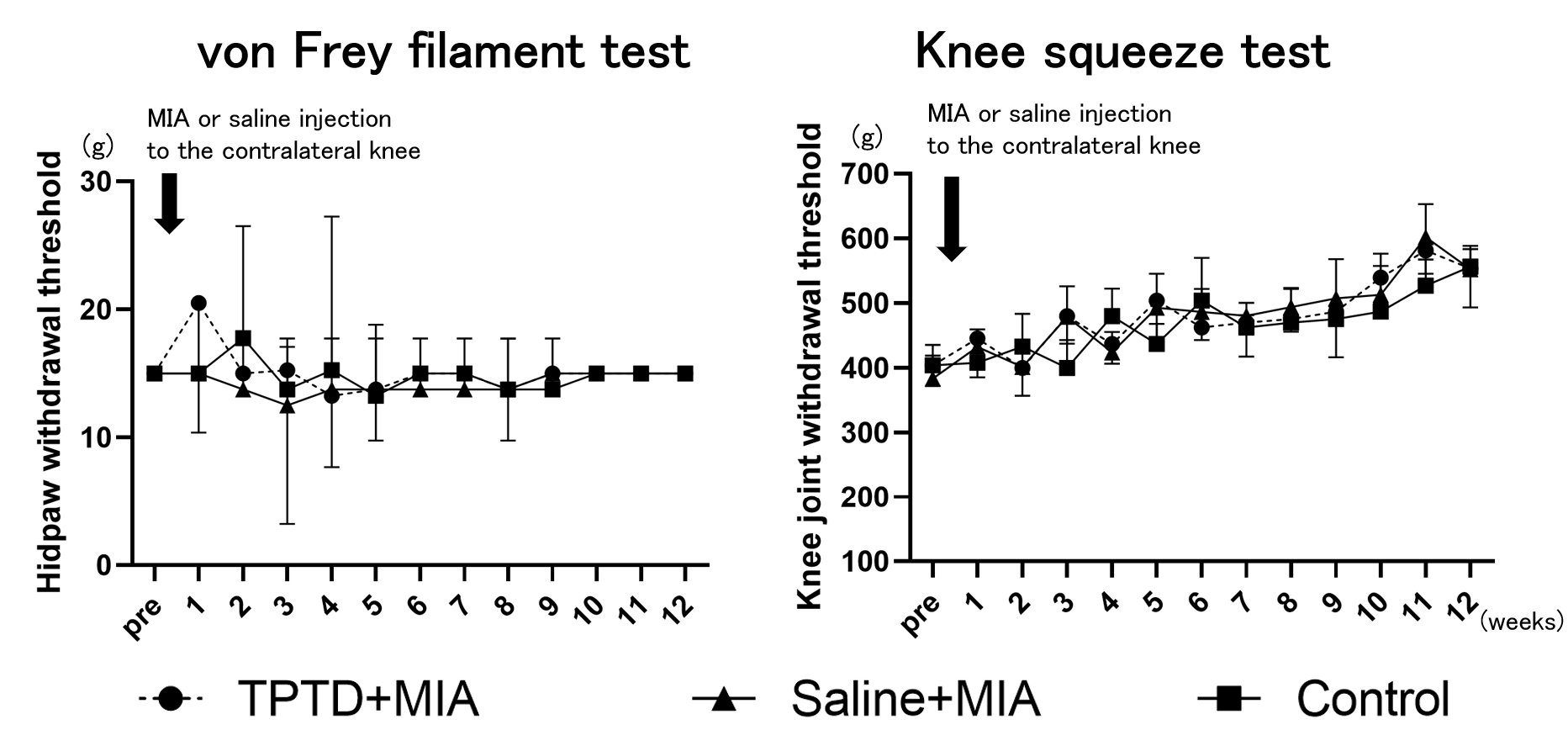


**Supplemental Figure. Effect of teriparatide and MIA on the mechanical threshold of the knee joint and hind paw on the contralateral side**

No significant differences in pain-related behaviors were observed among the three groups. Bars show the mean and 95% CI.
